# A Case of Suppressed Menstruation

**Published:** 1886-08-20

**Authors:** H. Christopher

**Affiliations:** St. Joseph, Missouri


### Southern Medical Record.

A MONTHLY JOURNAL OF PRACTICAL MEDICINE.

☞All Communications must be addressed to R. C. WORD, M. D., Managing Editor.

"*Quicquid Præcipies Esto Brevis.*"

VOL. XVI. ATLANTA, GA., AUGUST 20, 1886. No. 8

### ORIGINAL AND SELECTED ARTICLES.

### A CASE OF SUPPRESSED MENSTRUATION.

BY H. CHRISTOPHER, M. D., OF MISSOURI.

Miss A——, aged seventeen, past, while residing in Kentucky, suffered suppression while attending school in the Winter of 1884–'85, the last appearance taking place in the month of December, 1884. She was not in very good health at the time. The cessation continued until the school closed, in July, when she returned to Missouri, at which time she came under my care.

Up to this time she had continued in sufficient health to complete her studies at school, and return to her home in Missouri, with no *appearance* of deranged health but pallor of the face, thinness of flesh, and some general debility. The pallor was striking. It seemed to attract the observation of all who saw her, and surprise was expressed that she could be so well on her feet. Only her relations knew the real cause.

Besides the pallor, which was apparent to all, there was marked evidence of functional derangement of the liver, as shown by its characteristic pulse, furred tongue, and chronic inflammation of the pharyngeal mucous membrane, also characteristic of hepatic indigestion of months duration. Notwithstanding, the appetite was fair, and no meal was missed, though there was not a corresponding nutritious use of the food taken. The bowels were fairly regular. There was no pain complained of at any time, nor any troublesome symptoms, but a short, hacking cough, occurring almost unconsciously to the patient, so habitual had it become. It was more distressing to her friends than to herself. There was no pain in the loins, or bowels, or breasts, or head, at any periodical time, thus showing that the function of menstruation was in a kind of torpid state. At the time that treatment was begun she was eighteen years of age, and ceased to menstruate for a period of eight months. She had lost her mother some year before, and had been living with an aunt while at school, and was now with another, both of whom, of course, were anxious about her condition.

I placed her, at first, on small doses of calomel at night—generally about 1½ grains in powder—and continued the remedy until it had corrected the hepatic derangement. Once in the Fall of 1885 she had considerable fever, which kept her in bed and under more active treatment for about a week. When about the house again, 2-grain doses of quinine were given three times a day, as a tonic. In the course of a week, or more, the pulse again showed that the liver was still functionally deranged, and hence 1-grain doses of calomel at night were continued until the desired effect was produced.

In the Fall—November—of 1885 she was exposed to scarlet fever, her cousin, aged eight years, in an adjoining sleeping-room, having been taken with it. She escaped, however. The hacking cough, which had been relieved to a good degree, returned again, and she spat a little blood at one time. An examination of the lungs showed no disease there, and I so pronounced the condition of her lungs. I was satisfied in the beginning that the cough was not pneumonic.

I put her on the wine of coca, with the view of improving the condition of her digestive organs. This remedy was followed by the happiest effect, the cough entirely disappearing and returned no more. She was then placed on the four chlorides, and continued to take them for a month or more, when I put her on the phosphates of iron, quinine, strychnine, with lactopeptine. By this time the general health had improved considerably, and she had picked up a few pounds of flesh. Still there was no appearance of catamenia. In the Fall of 1885 I tried some of the so-called emmenagogues, but with no advantage whatever. I concluded, if general treatment, with iron and quinine as emmenagogues, would not cure her that nothing would; but when I saw an improvement in the general health under the use of calomel, quinine occasionally, and the four chlorides, I was confident that the catamenia would come in due time.

In the early Spring of this year—1886—her general health had improved so much that all pallor had disappeared, her face became full as in health, and the skin of a good complexion. She had gained fourteen pounds in flesh and bone, and looked as though nothing was ailing her. Still no catamenia; sometimes she would have some pain for a short time in the lower part of the abdomen, and in May there was a slight mucous discharge from the vagina, uncolored. This was hopeful. Later in the month there was a sense of fullness in the breasts, and some slight pain. This was more hopeful. On the 27th of May she left for her home in a neighboring city, and on the night following her arrival the catamenia appeared to her great surprise and joy.

The case seems of interest, because of the great length of time the suppression continued, and the absence of painful symptoms during the time. It is of interest also from the fact that the case was treated wholly on general principles. The emmenagogues were given on the solicitation of anxious relatives, but not on my own judgment. My confidence in restoring the functions lay wholly in the course I was pursuing—treating her on general principles. Though confidence and patience were sorely tried, I am gratified that they did not fail me under the pressure which comes on all physicians in such cases from anxious relatives.

*St. Joseph, Mo., July, 1886.*

